# Visualization of covalent intermediates and conformational states of proline utilization A by X-ray crystallography and molecular dynamics simulations

**DOI:** 10.1016/j.jbc.2025.110532

**Published:** 2025-07-28

**Authors:** David P. Buckley, Donald F. Becker, John J. Tanner

**Affiliations:** 1 Department of Biochemistry, University of Missouri, Columbia, Missouri, USA; 2 Department of Biochemistry, Redox Biology Center, University of Nebraska, Lincoln, Nebraska, USA; 3 Department of Chemistry, University of Missouri, Columbia, Missouri, USA

**Keywords:** crystal structure, molecular dynamics, flavoprotein, enzyme structure, proline dehydrogenase, aldehyde dehydrogenase, proline catabolism, bifunctional enzyme, substrate channeling

## Abstract

The bifunctional enzyme proline utilization A (PutA) catalyzes the two-step oxidation of L-proline to L-glutamate using proline dehydrogenase (PRODH) and L-glutamate-γ-semialdehyde dehydrogenase (GSALDH) domains. The two active sites are 42 Å apart and connected by a buried tunnel that is hypothesized to channel the intermediates Δ^1^-pyrroline-5-carboxylate (P5C) and/or L-glutamate-γ-semialdehyde (GSAL). Kinetic and conventional X-ray crystallography of PutA from S*inorhizobium meliloti* (SmPutA) were used to capture high resolution (1.47–1.88 Å) structures of states along the catalytic cycle, including a novel FADH^−^-proline covalent adduct in the PRODH site, the intermediate P5C bound noncovalently in the reduced PRODH active site, the covalent acyl-enzyme intermediate of the GSALDH reaction, and noncovalent complexes of GSAL and the final product L-glutamate in the GSALDH active site. The FADH^−^-proline covalent adduct resembles a stable species predicted from quantum mechanical electronic structure calculations of the PRODH reaction. The GSALDH domain complexes are consistent with conservation of substrate recognition and catalytic mechanism by the aldehyde dehydrogenase superfamily. The structure of reduced SmPutA with the P5C bound in the PRODH active site was used as the starting point for molecular dynamics simulations (21 × 2 μs trajectories). P5C diffuses from the PRODH active site into the tunnel in most of the trajectories, but rarely dissociates completely from the enzyme, consistent with previous kinetic evidence of a substrate channeling mechanism. The simulations also provide insight into protein conformational changes associated with substrate channeling, including the opening and closing of a conserved ion pair gate.

The proline catabolic pathway catalyzes the 4-electron oxidation of L-proline to L-glutamate (1). The pathway consists of two enzymes, which are linked metabolically by two intermediates (Fig. 1). The first enzyme, proline dehydrogenase (PRODH), catalyzes the FAD-dependent oxidation of L-proline to Δ^1^-pyrroline-5-carboxylate (P5C). The hydrolysis of P5C generates the second intermediate, L-glutamate-γ-semialdehyde (GSAL). The second enzyme of the pathway is GSAL dehydrogenase (GSALDH), which catalyzes the NAD^+^-dependent oxidation of GSAL to L-glutamate. GSALDH belongs to the aldehyde dehydrogenase (ALDH) superfamily and is also known as ALDH4A1.

**Figure 1:**
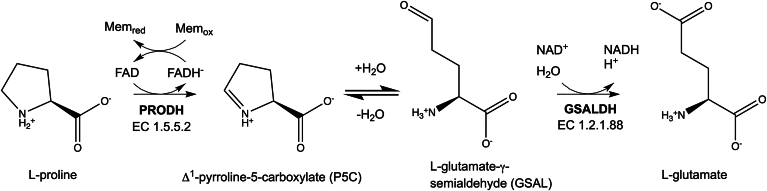
**Reactions of proline catabolism**.

Proline catabolic enzymes are highly conserved in bacteria and eukaryotes and are of considerable biomedical importance. PRODH in humans is regulated by p53 and is a tumor suppressor in some cellular contexts (2, 3). In other contexts, such as metastasizing breast cancer, PRODH promotes cancer and is a target of inhibitor discovery (4). Recent studies suggest that PRODH is also involved in the metabolism of naturally occurring thiazolidine carboxylates, which are reported to have therapeutic value by serving as nutrient and cysteine sources, and functioning as antioxidants and radical scavengers (5). GSALDH (a.k.a. ALDH4A1) is also regulated by p53 and in some cellular contexts may play a protective role against cell damage induced by the generation of intracellular reactive oxygen species (6). ALDHs have long been associated with cancer cell stemness (7), and high *ALDH4A1* gene expression has been reported in prostate cancer (8) and Hodgkin's lymphoma (9). Using a *Caenorhabditis elegans* model, ALDH4A1 was identified as a predictive biomarker for age-related changes in muscle health and may be a critical component of muscle aging (10). ALDH4A1 is significantly elevated in the plasma of atherosclerosis-prone mice and atherosclerotic human tissue, and anti-ALDH4A1 antibodies have potential therapeutic value in cardiovascular disease (11).

In some bacteria, PRODH and GSALDH are combined into a bifunctional enzyme known as proline utilization A (PutA) (12). PutAs have been implicated in bacterial virulence mechanisms (13, 14, 15, 16, 17). PutA polypeptide chains range from about 1000 to over 1300 residues, with the PRODH and GSALDH active sites located in the N and C terminal halves of the protein, respectively.

Crystal structures of PutAs have revealed a conserved multidomain architecture (Fig. 2). The PRODH active site is located in a (βα)_8_ barrel, while the GSALDH module features Rossmann NAD^+^-binding and catalytic domains, which are both characteristic of the ALDH superfamily. The PutA fold also includes several other domains that are thought to help maintain a specific three-dimensional relationship of the two active sites, including a spatial separation of 42 Å and intervening substrate-channeling tunnel. PutAs serve as excellent systems for studying the structural basis and mechanism of substrate channeling (18).

**Figure 2:**
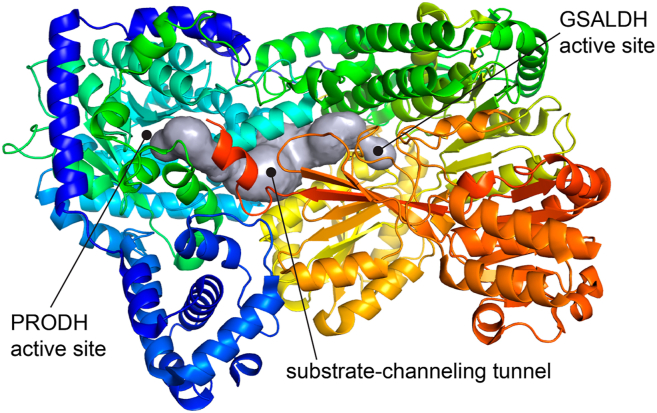
**Fold of SmPutA, as seen in the complex with the product L-glutamate (**PDB: 9BBO**).** The polypeptide chain is colored in a *rainbow* scheme with *blue* at the N terminus and red at the C terminus. The tunnel was calculated with the MOLEonline server (79). SmPutA, proline utilization A from *Sinorhizobium meliloti*; PDB, Protein Data Bank.

Herein, we describe the results of a combined X-ray crystallography and molecular dynamics (MD) study of PutA from *Sinorhizobium meliloti* (SmPutA). Kinetic and conventional X-ray crystallography enabled structural characterization of six species along the reaction pathway starting with a novel covalent FADH^−^-proline adduct, the intermediate P5C bound in the reduced PRODH active site, a secondary proline/P5C binding site in the PRODH domain, a noncovalent complex with the substrate GSAL bound in the GSALDH active site, the covalent acyl-enzyme intermediate of the GSALDH reaction, and the product L-glutamate bound in the GSALDH active site. The P5C complex obtained *via* kinetic X-ray crystallography was used to launch MD simulations designed to visualize substrate channeling *in silico*. Together, the crystal structures provide models of distinct states along the catalytic cycle, while the MD simulations provide insight into the dynamics of substrate channeling.

## Results

### Structure of a covalent FADH^−^-proline adduct

**Figure 3:**
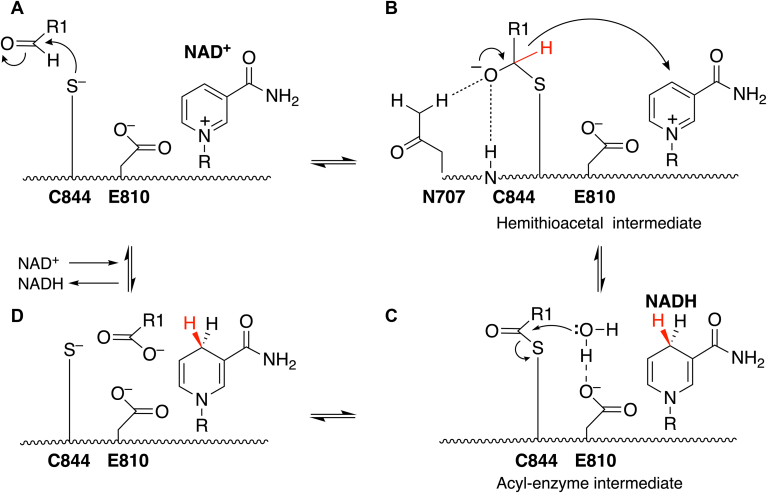
**Mechanism of ALDH superfamily enzymes**. Residue numbers refer to SmPutA. The color *red* is used to highlight the stereospecificity of hydride transfer. SmPutA, proline utilization A from *Sinorhizobium meliloti*; ALDH, aldehyde dehydrogenase.

Kinetic protein crystallography approaches were used to capture intermediates and conformational states along the PutA reaction cycle. The term “kinetic crystallography” generally refers to experiments in which turnover is induced within the crystal, and various strategies are used to capture transient species (19, 20). Often, the time scale of the reaction of interest is slowed using low temperature, pH, suboptimal substrates, or site-directed variants. We used the latter approach with two mutations targeting conserved catalytic residues in the GSALDH active site, Cys844 and Glu810 (Fig. 3). Cys844 attacks the carbonyl of GSAL in the first step of the mechanism (Fig. 3*A*); the mutation of Cys844 to Ser disables the GSALDH activity of PutA. Glu810 facilitates hydrolysis of the acyl-enzyme intermediate (Fig. 3*C*), and its mutation to Ala is expected to slow the hydrolysis step, allowing the acyl-enzyme intermediate to build up.

**Figure 4:**
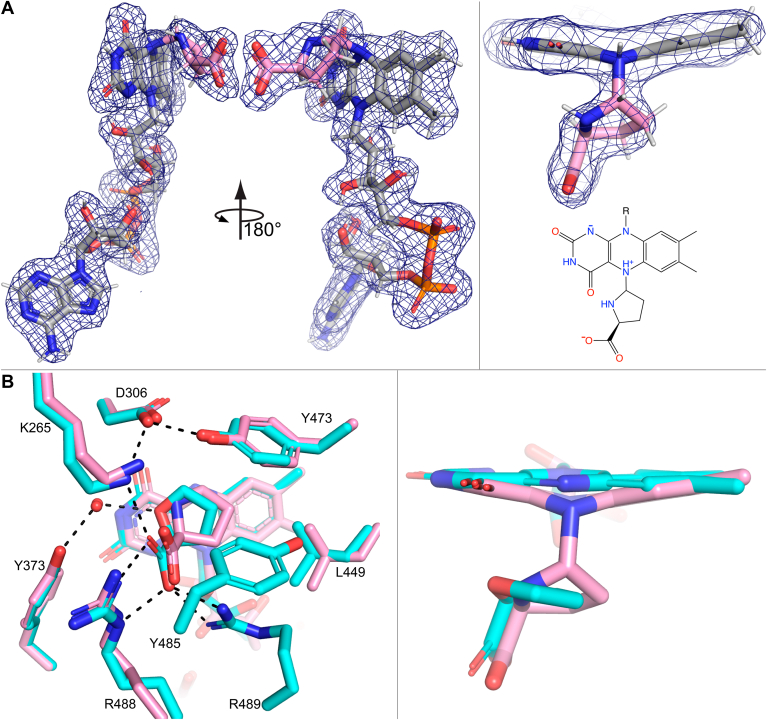
**Structure of the FADH^−^-proline covalent adduct**. *A*, electron density for the covalent adduct (polder omit, 4σ). *B*, comparison of the active sites of the FADH^−^-proline covalent adduct (*pink*) and the noncovalent complex with the proline analog THFA (*cyan*, PDB: 5KF6). *Dashes* represent electrostatic interactions in the THFA complex. THFA, S-(−)-tetrahydro-2-furoic acid; PDB, Protein Data Bank

Kinetic crystallography experiments with C844S unexpectedly revealed a new covalent adduct of the FAD. Catalytic cycling of the PRODH reaction was performed *in crystallo* by soaking crystals of the C844S variant of SmPutA in 40 mM L-proline, 1 mM Coenzyme Q_1_, and 1 mM NAD^+^. The soaking time was varied over a wide range as described in Experimental procedures, followed by flash-cooling the crystal in liquid nitrogen. Surprisingly, the structure determined after 24 h of catalytic cycling (1.88 Å resolution, Table 1) showed strong electron density evidence for a species covalently bound to the N5 of the FAD (Fig. 4*A*). This feature was more prominent in chain B than chain A. The electron density is consistent with L-proline or P5C covalently linked to the FAD N5. Based on a recent quantum mechanics-molecular mechanics study of the PRODH reaction, we have modeled this species as L-proline bonded to reduced FAD (21). The occupancy of the FADH^−^-proline adduct was set to 1.0 after trial refinement calculations suggested full occupancy.

**Table 1:** X-ray diffraction data processing and refinement statistics

Parameter	FADH^−^-Pro	GSAL	Acyl-enzyme	L-Glutamate
Beamline	APS 24-ID-E	ALS 4.2.2	APS 24-ID-C	APS 24-ID-E
Space group	*P*2_1_	*P*2_1_	*P*2_1_	*P*2_1_
Unit cell parameters (Å,°)	*a* = 101.60 *b* = 102.57 *c* = 127.20 *β* = 106.47	*a* = 100.87 *b* = 101.82 *c* = 126.03 *β* = 106.53	*a* = 101.78 *b* = 103.12 *c* = 127.92 *β* = 106.51	*a* = 101.38 *b* = 102.43 *c* = 127.60 *β* = 106.38
Resolution (Å)	122.0–1.88 (1.92–1.88)	48.3–1.47 (1.58–1.47)	103.1–1.74 (1.77–1.74)	102.4–1.50 (1.53–1.50)
*R*_pim_(*I*)^a^	0.042 (0.486)	0.031 (0.723)	0.094 (1.052)	0.033 (0.459)
Mean I/σ^a^	12.5 (1.4)	12.6 (0.80)	4.5 (0.3)	17.5 (1.9)
CC_1/2_^a^	0.998 (0.534)	0.998 (0.357)	0.986 (0.331)	0.998 (0.627)
Completeness (%)^a^	97.2 (87.8)	97.7 (78.6)	96.2 (60.6)	97.2 (95.0)
Multiplicity^a^	7.1 (6.5)	4.9 (2.3)	3.8 (3.0)	6.1 (3.9)
No. of non-H atoms				
Protein	17,646	17,808	17,650	18,349
FAD	53	106	106	106
NAD	88	132	88	N/A
PRODH ligands	61	N/A	N/A	N/A
GSALDH ligands	N/A	18	9	20
Water	1279	1734	1141	1982
*R*_cryst_/*R*_free_^b^	0.170/0.206	0.180/0.207	0.211/0.249	0.169/0.191
rmsd bonds (Å)	0.010	0.009	0.010	0.006
rmsd angles (°)	1.060	1.072	1.105	0.850
Ramachandran plot^c^				
Favored (%)	97.46	97.59	97.59	98.11
Outliers (%)	0.33	0.04	0.12	0.04
Clashscore^c^^d^	2.17 (99%)	2.33 (99%)	1.97 (99%)	1.48 (99%)
MolProbity score^c^^d^	1.10 (100%)	1.10 (100%)	1.05 (100%)	0.89 (100%)
Average B (Å^2^)				
Protein	32.7	21.9	37.3	21.7
FAD	32.1	18.8	38.7	17.2
NAD	29.1	15.8	33.5	N/A
PRODH ligands	30.4	N/A	N/A	N/A
GSALDH ligands	N/A	20.7	37.3	18.1
Water	34.7	27.0	36.9	26.8
PDB ID	9C34	9C36	9C35	9BBO

a Values for the outer resolution shell of data are listed in parentheses.

b 5% test set.

c From MolProbity.

d Percentile ranks are listed in parentheses.

The electron density clearly defines the conformation of the adduct (Fig. 4*A*). The covalent adduct links the N5 of the FAD to C5 of L-proline. The N5-C5 bond length refined to 1.66 Å, which is rather long for an N-C bond but consistent with the aforementioned quantum mechanics-molecular mechanics study (21). The FAD exhibits strong butterfly bending of the isoalloxazine with a bend angle of ∼20° (*si* face convex). Butterfly bending of the FAD in PRODH is diagnostic of the 2-electron reduced state (22, 23, 24, 25, 26, 27, 28). The pyrrolidine ring of proline is puckered.

The pose and interactions of the covalently bound L-proline are reminiscent of the substrate L-proline in the Michaelis complex. A good model for the Michaelis complex is SmPutA complexed with the proline analog inhibitor S-(−)-tetrahydro-2-furoic acid (THFA, Protein Data Bank (PDB): 5KF6). The two structures are compared in Fig. 4*B*. Interactions common to both complexes include the ion pairs with Arg488 and Lys265, and the nonpolar contacts of the pyrrolidine ring with Tyr473 and Leu449 (Fig. 4*B*). All these interacting residues are highly conserved in PRODHs from bacteria to eukaryotes.

A few differences from the SmPutA-THFA complex are also observed (Fig. 4*B*). For example, the adduct is shifted by 0.5 to 0.8 Å toward the hydrophobic pocket so it is more centered over the middle ring of the isoalloxazine. This shift takes the amino group of the adduct out of hydrogen bonding distance to a conserved water, which hydrogen bonds to the heteroatom of THFA. THFA forms an additional ion pair with conserved Arg489; this residue has very weak electron density in the adduct structure and is presumably conformationally disordered. Similarly, Tyr485 packs against the THFA ring but is disordered in the adduct structure. Altogether, the active site in the adduct structure is partially disassembled compared to the Michaelis complex.

### Noncovalent P5C complex and discovery of a secondary proline/P5C binding site

**Figure 5:**
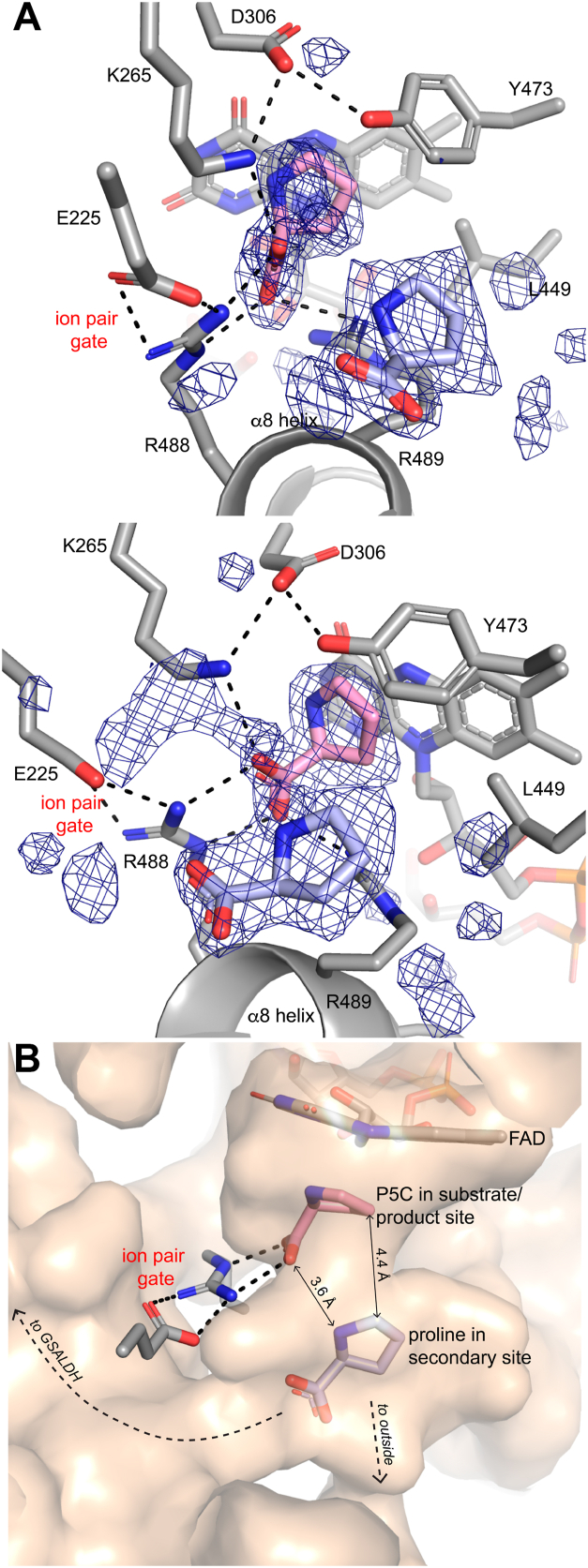
**P5C product and secondary proline/P5C site.***A*, two views of electron density for P5C bound noncovalently at the *si*-face of the FAD (*pink*) and proline bound in the secondary binding site (*light blue*). The ion pair gate, which is closed, is noted. The mesh represents a polder omit map (4.5σ) drawn with a buffer of 10 Å around the ligands. *B*, surface representation of the active site with P5C and proline bound. The surface represents internal cavities and tunnels visualized with PyMOL (76). P5C, Δ^1^-pyrroline-5-carboxylate.

As noted in the previous section, the covalent FADH^−^-proline adduct was modeled only in chain B. In chain A, electron density for the adduct was weak; however, two electron density features consistent with either proline or P5C bound noncovalently in the PRODH active site were observed (Fig. 5*A*). One of these features is consistent with a proline-like molecule bound noncovalently next to the *si*-face of the reduced FAD and was interpreted as representing a newly produced P5C molecule (occupancy of 0.73). The other site appears to be a heretofore uncharacterized secondary binding site and was interpreted as either proline entering the active site or P5C leaving (occupancy of 0.87). Proline has been modeled into the secondary site.

The P5C product molecule occupies the substrate site of the Michaelis complex and its interactions with the enzyme resemble those of proline analog, THFA, including ion pairs with Arg488, Arg489, and Lys265, and nonpolar contacts with Tyr473 and Leu449 (Fig. 5*A*). The proline molecule in the secondary site is ∼4 Å from the P5C molecule and contacts the α8 helix (Fig. 5*B*). The secondary proline forms no hydrogen bonds and appears to be stabilized mainly by shape complementarity with a cavity that connects to both the outside environment and the substrate channeling tunnel.

### Structure of the noncovalent GSAL complex

Kinetic crystallography was also used to capture a structure of SmPutA variant C844S complexed with GSAL and NAD^+^. Catalytic cycling was performed by soaking crystals in 40 mM L-proline, 1 mM CoQ_1_, and 1 mM NAD^+^ for ∼1 min before flash-cooling the crystal in liquid nitrogen. Cys844 is the catalytic cysteine of SmPutA (Fig. 3) and the use of C844S disabled the GSALDH reaction, allowing GSAL to build up in the crystal. The structure was determined at 1.47 Å resolution (Table 1).

**Figure 6:**
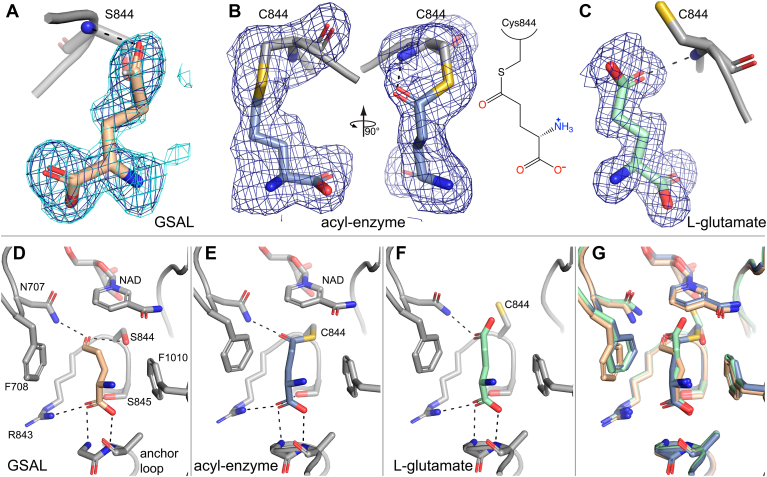
**Ligands captured in the GSALDH active site**. *A*, electron density for noncovalently bound GSAL in chain B (Q = 0.72). Polder omit maps at 4σ (*blue*) and 3.5σ (*aquamarine*) are shown. *B*, electron density (polder 4σ) for the acyl-enzyme intermediate in chain A (Q = 0.83). *C*, electron density (polder 4σ) for the product L-glutamate (Q = 1.0). *D*, pose and interactions of GSAL. *E*, pose and interactions of the acyl-enzyme intermediate. *F*, pose and interactions of L-glutamate. *G*, superposition of all three structures. Ligand coloring is the same as in the other panels. GSALDH, L-glutamate-γ-semialdehyde dehydrogenase.

Electron density maps suggested the presence of a ligand bound in the GSALDH active site (Fig. 6*A*). GSAL was modeled with refined occupancies of 0.75 in chain A and 0.72 in chain B. GSAL is stabilized by several interactions with the enzyme (Fig. 6*D*). The aldehyde carbonyl accepts hydrogen bonds from conserved Asn707 and the backbone of Ser844. These interactions define the oxyanion hole, a conserved feature of substrate recognition by ALDH superfamily enzymes (29, 30). The carbonyl also hydrogen bonds directly with Ser844, an interaction that would be absent in the WT enzyme. The aliphatic chain of GSAL is flanked by Phe708 and Phe1010, which form the “aromatic box”, another conserved element of substrate recognition in the ALDH superfamily. The carboxylate of GSAL forms electrostatic interactions with the backbone of the anchor loop and side chains of Ser845 and Arg843; these interactions are also observed with proline and hydroxyproline inhibitors of SmPutA (31).

### Structure of the acyl-enzyme covalent intermediate

The structure of the acyl-enzyme intermediate was captured by soaking crystals of the E810A variant in 40 mM L-proline, 1 mM Coenzyme Q_1_, and 1 mM NAD^+^ for 24 h and then flash-cooling the crystal in liquid nitrogen. The acyl-enzyme intermediate appears in the third step of the ALDH mechanism, after hydride transfer from the hemithioacetal intermediate to NAD^+^ to form NADH (Fig. 3). Glu810 is the conserved glutamate in the GSALDH active site that facilitates hydrolysis of the acyl-enzyme intermediate, and mutation to Ala is expected to slow the hydrolysis step, allowing the acyl-enzyme intermediate to build up. This strategy has been used to trap the acyl-enzyme intermediate of glyceraldehyde 3-phosphate dehydrogenase (32).

The structure of the acyl-enzyme intermediate was resolved at 1.74 Å resolution (Table 1). Electron density clearly showed the presence of a covalent modification of Cys844 in chain A (Fig. 6*B*). The electron density is consistent with the acyl-enzyme intermediate, which was modeled with occupancy of 0.83. The covalently bound GSAL is stabilized by the same interactions described above for noncovalent GSAL, involving the oxyanion hole, aromatic box, and anchor loop (Fig. 6*E*).

Electron density for the coenzyme, either NAD^+^ or NADH, was also observed (occupancy of 1.0 in both chains). The coenzyme adopts the pose associated with NAD^+^ prior to hydride transfer (hydride transfer pose) and is distinct from the pose associated with NADH in which the nicotinamide riboside has withdrawn from the catalytic pocket (33). Given that the crystal was soaked with excess NAD^+^, it is possible that NADH dissociated after formation of the acyl-enzyme and was replaced by NAD^+^. Alternatively, the bound coenzyme may be NADH, and use of the E810A variant and/or the crystalline environment inhibited coenzyme retraction. Given that the coenzyme is in the pose typical for NAD^+^, we have modeled it as NAD^+^. We note that the electron density did not indicate puckering of the nicotinamide ring as would be expected for NADH, but this feature is challenging to discern at 1.74 Å resolution.

### Structure of the product complex with L-glutamate

The structure of SmPutA complexed with the product L-glutamate was determined at 1.50 Å resolution (Table 1). The complex was prepared by soaking WT crystals with 325 mM L-glutamate. Electron density for the product is strong in both chains in the asymmetric unit, enabling modeling of the ligand at occupancy of 1.0 (Fig. 6*C*).

L-glutamate adopts an extended conformation, with its backbone interacting with the anchor loop, the side chain carboxylate in the oxyanion hole, and the aliphatic chain in the aromatic box (Fig. 6*F*). Cys844 in this complex exhibits χ1 of −69°, instead of the nucleophilic attack rotamer (55°), to avoid steric clash with the side chain carboxylate of the product. We note that the pose and interactions of glutamate in SmPutA are very similar to those in structures of monofunctional ALDH4A1-glutamate complexes reported previously (PDB: 2BHQ, 3V9K) (34, 35).

The interactions of the product are similar to those of GSAL in the noncovalent complex and the acyl-enzyme intermediate. For example, the interactions with the oxyanion hole, aromatic box, and anchor loop are virtually identical in the three structures (Fig. 6, *D*–*F*). Also, the carbonyl O-atoms of all three complexes are nearly superimposed (Fig. 6*G*). One difference is that the carbonyl group of noncovalent GSAL is rotated away from the catalytic residue (residue 844), apparently causing subtle rotations of Phe708 and Asn707 (Fig. 6*G*).

### MD simulations of the product complex of reduced SmPutA with P5C

MD simulations were performed to gain insight into the dynamics of substrate channeling. The starting model for the simulations was derived from chain A of the crystal structure of C844S after soaking for 24 h in 40 mM L-proline, 1 mM Coenzyme Q_1_, and 1 mM NAD^+^ (PDB: 9C34). In chain A, the PRODH active site contains a P5C molecule noncovalently bound next to the *si*-face of the reduced FAD and L-proline in the secondary site. The GSALDH active site contains NAD^+^ with an associated Mg^2+^ ion. This structure is interpreted as representing the P5C product complex prior to substrate channeling. L-proline in the secondary site was omitted for the simulation. Twenty-one MD trajectories, each 2 μs long, were performed resulting in a total of 42 μs of simulation time. This is the first MD simulation of any PutA.

**Figure 7:**
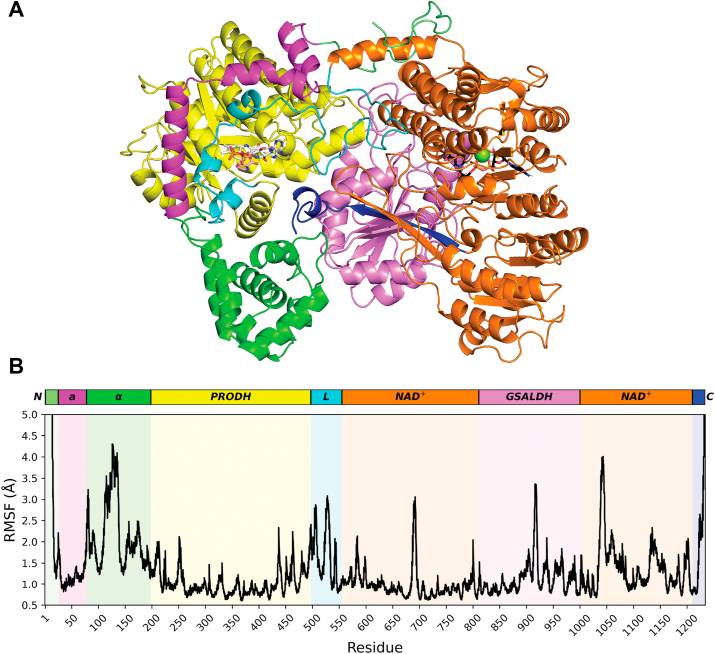
**Average backbone RMSF (root-mean-square fluctuation) of SmPutA for combined 42 μs of simulation data**. *A*, domain architecture of SmPutA colored according to the diagram in *panel B*. FADH_2_ and NAD^+^ are shown in *white* and *black sticks*, respectively. The *green sphere* represents a Mg^2+^ ion bound to NAD^+^. *B*, average per-reside backbone RMSF. SmPutA, proline utilization A from *Sinorhizobium meliloti*; RMSF, root-mean-square fluctuation.

The MD simulations indicated flexible structural elements of SmPutA. The average backbone root-mean square fluctuation (RMSF) shows that the most flexible parts of the enzyme are the N and C termini and loops between secondary structure elements, as expected (Fig. 7). The C terminus is notable because it forms a lid that covers part of the substrate channeling tunnel in crystal structures of SmPutA and other PutAs. Also, the RMSFs are elevated throughout the entire α-domain (residues 85–194). The α-domain is a structural domain that contacts the two catalytic units of the enzyme. This domain exhibits regions of conformational disorder in PutA crystal structures, consistent with its high flexibility in the MD simulations. In contrast, residues of the substrate channeling tunnel exhibit low RMSF, consistent with the tunnel being buried in the protein interior. For example, residues 373 to 385 and 824 to 842 form two α-helices that line the middle part of the tunnel and have RMSFs of less than ∼1 Å (Fig. 7).

The simulations provide insight into hydration of the substrate-channeling tunnel, a feature not immediately evident from crystal structures. For example, only 60 to 80 water molecules are resolved in the tunnel of one protomer of the 1.50 Å resolution glutamate complex crystal structure (PDB: 9BBO) (Fig. S1). Almost all these crystallographic water molecules form hydrogen bonds with residues of the tunnel walls, so the interior of the tunnel appears empty in the crystal structure. In contrast, the time- and ensemble-averaged number of tunnel water molecules from MD is ∼280 (Fig. S2). Hydration of the tunnel is dynamic, as expected, and the number of tunnel waters fluctuates between about 250 and 325. The number of tunnel waters from MD is considerably higher than an earlier estimate of ∼170 based on cavity volume calculations of a PutA crystal structure (36).

**Figure 8:**
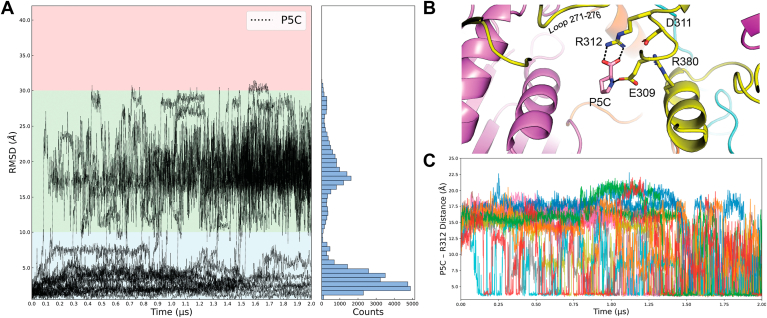
**P5C dynamics during MD simulations**. *A*, RMSD of P5C for 20 replicate simulations, calculated from its starting position in the PRODH site. The single simulation in which P5C completely dissociated from the enzyme is omitted for clarity. The *y*-axis is color-coded to indicate regions near PRODH (*blue*), the substrate channeling tunnel (*green*), and GSALDH (*red*). Binned P5C RMSD values from the combined replicate simulations are also provided. *B*, MD snapshot of P5C at ∼17.5 Å along the channeling pathway. Residues within 5 Å of P5C are shown in *sticks*. SmPutA is colored by its domain architecture (PRODH: *yellow*, GSALDH: *pink*). *C,* time evolution of the P5C-Arg312 interaction distance for 15 simulations where P5C was released into the tunnel. PRODH, proline dehydrogenase; SmPutA, proline utilization A from *Sinorhizobium meliloti*; GSALDH, L-glutamate-γ-semialdehyde dehydrogenase; P5C, Δ^1^-pyrroline-5-carboxylate; MD, molecular dynamics.

The location of the P5C was tracked to gain insight into the dynamics of substrate channeling. The RMSD of P5C from its initial position in the PRODH site was calculated for each of the 21 replicate MD simulations (Fig. 8*A*). In six of the simulations, P5C remained in the PRODH active site, which is operationally defined as within 10 Å of the P5C starting point (blue zone in Fig. 8*A*). In the other 15 trajectories, P5C exited the PRODH active site and entered the tunnel (green zone in Fig. 8*A*). P5C dissociated completely from the enzyme in one simulation (omitted in Fig. 8*A* for clarity). These results are consistent with kinetic data showing that PutAs protect the intermediate from release into the bulk solvent and support the assertion that the tunnel observed in PutA crystal structures functions in substrate channeling.

The simulations suggest that certain locations in the tunnel may be docking sites en route to the GSALDH active site. The histogram of P5C RMSD shows a large peak at 0 to 5 Å from the initial location, corresponding to P5C in the PRODH active site before diffusion into the tunnel (Fig. 8*A*). The more interesting part of the histogram is the region between 10 Å and 30 Å, which corresponds to P5C in the tunnel. The distribution in the tunnel is not uniform as P5C accumulates at ∼17.5 Å along the channeling pathway. This location is centered between two α-helices (residues 373–385 and 824–842) that form the main walls of the tunnel (Fig. 8*B*). Flexible loops 271 to 276 and 308 to 312 of PRODH extend into the tunnel and form the “roof” of the binding site (Fig. 8*B*). P5C in this location can form an ion pair with Arg312, as well as a hydrogen bond with Glu309 (Fig. 8*B*). The Arg312-P5C ion pair was tracked by monitoring the distance between NZ of Arg312 and the carbonyl C-atom of P5C (Fig. 8*C*). This interaction is formed in all the channeling trajectories, as evidenced by interaction distances < 5 Å, and routinely persists for 50 to 100 ns.

The MD simulations provide insight into the initial step of substrate channeling, when P5C exits the PRODH active site and enters the tunnel. This analysis focused on the Arg488-Glu225 ion pair, which has long been considered the gate of the PRODH active site (24, 26, 36, 37). The gate is closed (ion pair formed) in all structures of PutAs complexed with proline analogs, and opening of the gate has been hypothesized to accompany release of the P5C into the tunnel. In the starting configuration of the MD simulations, P5C occupies the proline/P5C substrate/product site and the gate is closed (Fig. 5). The status of the gate was tracked by monitoring the distance between the CZ atom of Arg488 and the CD atom of Glu225.

**Figure 9:**
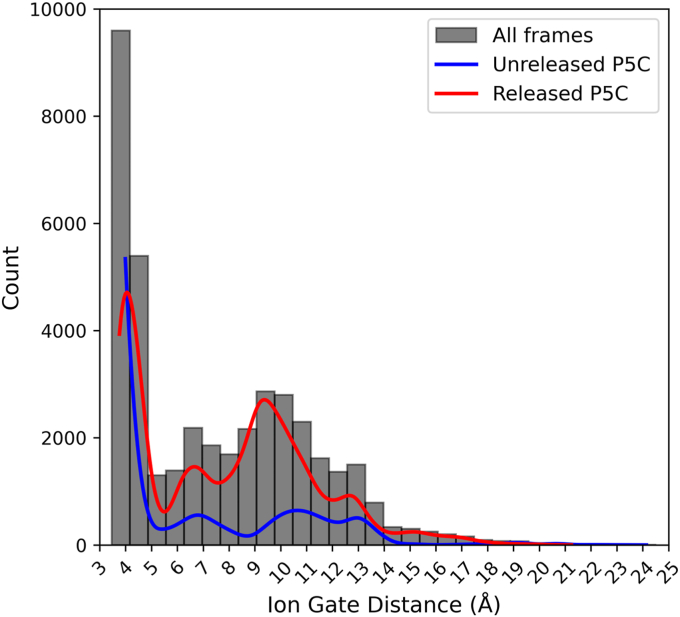
**Distribution of the ion-pair gate status.** Binned pairwise interaction distances between Arg488 and Glu225 and for all combined simulation frames as a histogram (*black*). The *blue curv*e shows the gate distance distribution for the subset of frames in which P5C remained in the PRODH active site. The *red curve* shows the gate distance distribution for the subset of frames in which P5C left the PRODH active site and entered the tunnel. PRODH, proline dehydrogenase; P5C, Δ^1^-pyrroline-5-carboxylate.

The distribution of gate distances implies a range of states (Fig. 9). The major peak at 3 to 5 Å corresponds to closed conformations. The distribution also shows semiopen states with distances of 5 to 8 Å and fully open states characterized by interaction distances of 8 to 18 Å. The open states are more frequently sampled in the trajectories in which P5C was released into the tunnel (Fig. 9, red curve) than in those where P5C remained in the active site (Fig. 9, blue curve), suggesting that opening of the gate is associated with substrate channeling.

**Figure 10:**
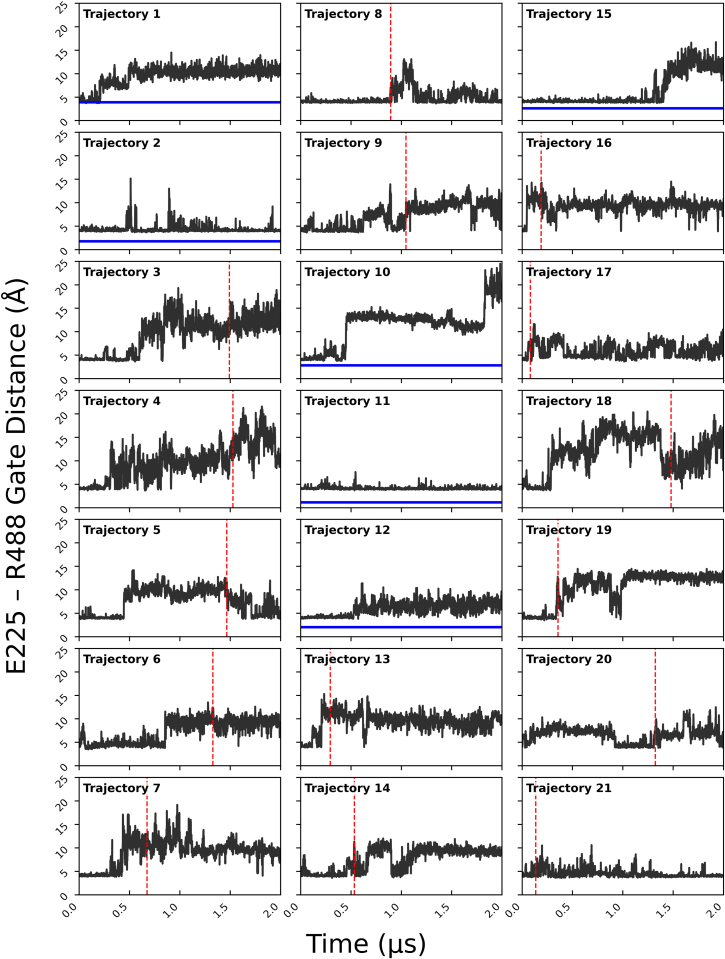
**Time evolution of the Arg488-Glu225 ion pair gate distance for 21 replicate simulations**. For the six trajectories where P5C was not released, the *blue line* represents the time average of P5C RMSD. For trajectories where P5C is released, the *vertical red dashed line* indicates the time of release. P5C fully dissociated from the enzyme in trajectory 16. P5C, Δ^1^-pyrroline-5-carboxylate.

To better understand the contribution of the gate to substrate channeling, we analyzed the temporal relationship between gate opening and P5C release. The gate is highly dynamic and exhibits a wide range of open states with various lifetimes. At one extreme, two trajectories showed the gate was predominantly closed and any open states were very short-lived (Fig. 10, #2, #11); P5C remained in the PRODH active site in these cases. In contrast, highly open states (≥10 Å) that persisted for over 1 μs were observed in most of the trajectories (#1, #3–7, #9–10, #13, #16, #18–19); P5C entered the tunnel in most, but not all, of these trajectories. The vertical dashed lines in Fig. 10 indicate when P5C moved into the tunnel. In all cases, the gate was open, usually in a long-lived fully open state (*e.g.*, #3), when P5C moved into the tunnel. We note that P5C never returned to the PRODH active site, despite the gate remaining open. Also, sometimes the gate closed after P5C entered the tunnel (#5, #8). Altogether, the MD simulations suggest that opening of the gate is necessary, but not sufficient, for release of P5C into the tunnel and hence substrate channeling.

## Discussion

**Figure 11:**
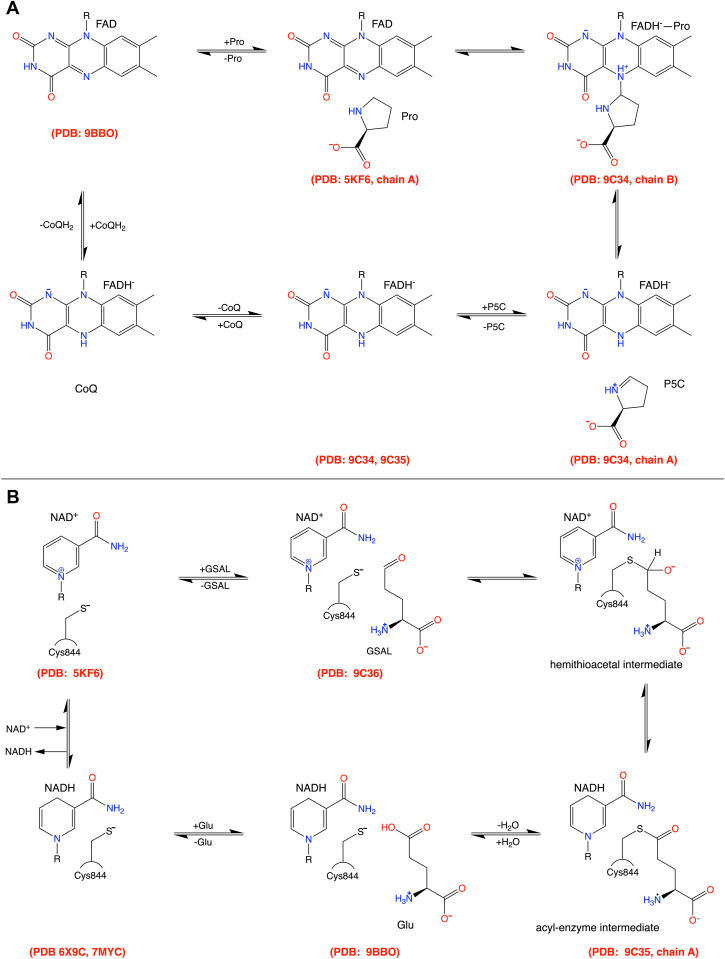
**Summary of the available X-ray crystal structure models for the catalytic cycle of PutA**. PDB IDs for relevant crystal structures are listed in *red*. *A*, steps in the PRODH reaction cycle. *B*, steps in the GSALDH reaction cycle. PRODH, proline dehydrogenase; PutA, proline utilization A; GSALDH, L-glutamate-γ-semialdehyde dehydrogenase; PDB, Protein Data Bank.

The crystal structures reported here are new for the PutA protein family and fill in several missing states of the PutA catalytic cycle (Fig. 11). The catalog of SmPutA crystal structures now provides an almost complete set of models for the major intermediates and conformations. In the PRODH part of the cycle (Fig. 11*A*), the new structures include a covalent FADH^−^-proline adduct and the noncovalent FADH^−^-P5C complex. In the GSALDH part of the cycle, we described a noncovalent GSAL complex, the acyl-enzyme intermediate, and the glutamate product complex (Fig. 11*B*). In addition, the structures of the noncovalent GSAL complex and the acyl-enzyme intermediate provide views of the ligand-free reduced PRODH active site, while the glutamate complex shows the ligand-free oxidized PRODH active site. An outstanding challenge in PutA structural biology is to capture a complex of the reduced enzyme with a biologically-relevant quinone. Although a structure of reduced PutA from *Geobacter sulfurreducens* complexed with menadione bisulfite is available (PDB: 4NMF), the presence of the anionic sulfite group is atypical for biological quinones and may have induced structural artifacts (24).

The FADH^−^-proline covalent adduct is similar to an intermediate predicted from electronic structure calculations of the PRODH reaction by Yildiz (21). The calculations predicted a stable species (“PC2”) occurring after hydride transfer to the FAD and before release of P5C. In the optimized geometry, the distance between N5 of the reduced isoalloxazine and C5 of proline is 1.66 Å “suggesting that there is a strong dipole-dipole interaction between N5 and C5 atoms”. Yildiz notes that the distance of 1.66 Å does not suggest a full covalent bond but could indicate a strong coordination interaction. In our crystal structure determined from kinetic crystallography, we observed strong electron density indicating butterfly bending of the isoalloxazine ring, implying the FAD is 2-electron reduced, and a bond between the FAD N5 and proline C5 atoms. Use of quantum mechanical restraints (QMR) during crystallographic refinement resulted in a N5-C5 bond length of 1.67 Å, essentially identical to Yilidz's prediction. Alignment of our crystal structure and the Yildiz model shows excellent agreement (Fig. S3). We conclude that we experimentally captured a new state in the PRODH reaction whose existence was postulated from electron structure calculations.

The FADH^−^-proline covalent adduct also resembles the covalent FAD adduct obtained with thiazolidine-2-carboxylate (T2C) (28). T2C and the related compound thiazolidine-4-carboxylate are alternative substrates for PRODH (5). Cocrystallization of SmPutA with T2C resulted in the growth of colorless crystals, consistent with T2C being a substrate. Unexpectedly, the structure revealed that the N5 atom of the reduced FAD was covalently bonded to the C5 atom of T2C (Fig. S4). As with the FADH^−^-proline covalent adduct, the active site of the T2C adduct largely resembles the Michaelis complex, but is partially disassembled, with Tyr485 exhibiting conformational disorder. At the time, T2C was considered to be a mechanism-based inactivator, albeit a very slow one, characterized by a kinetic timescale of hours. An inactivation mechanism was proposed in which the C5 atom of T2C is oxidized, resulting in a sulfur-stabilized carbocation, which is attacked by the N5 of the reduced FAD. Considering the FADH^−^-proline covalent adduct reported here, an alternative interpretation is that the T2C adduct represents a reaction intermediate.

We also captured the covalent acyl-enzyme intermediate of the GSALDH reaction. This structure is the first for PutA and the ALDH4A1 enzyme family; however, precedent is found by wider consideration of the entire ALDH superfamily. The first crystal structure of an acyl-enzyme intermediate for the superfamily was determined about 20 years ago for nonphosphorylating NADP-dependent glyceraldehyde 3-phosphate dehydrogenase (GAPN, PDB: 2ESD, 2QE0) (32). The GAPN and SmPutA acyl-enzyme intermediates share several common features, including trigonal planar geometry, hydrogen bonding of the carbonyl to both the side chain of a conserved Asn and the main chain of the catalytic cysteine, packing of the middle of the substrate into the aromatic box, and interaction of the distal end of the intermediate with the anchor loop. These results are consistent with conservation of substrate recognition and catalytic mechanism among ALDH superfamily enzymes.

The noncovalent GSAL complex is consistent with the Michaelis complex in some, but not all, respects. NAD^+^ is positioned as expected in the hydride transfer pose (Fig. 6*D*). Ser844 mimics the nucleophilic attack conformation of Cys844 by adopting χ1 near 55°, as expected for the Michaelis complex. Also, GSAL interacts with conserved recognition elements of the ALDH superfamily, including the oxyanion hole, aromatic box, and anchor loop. In contrast, the orientation of the carbonyl bond vector relative to the nucleophile (Ser844 hydroxyl representing Cys844 S^-^) is not optimal. The Burgi-Dunitz angle (Nu-C-O) measured from the structure is 61°, compared to the optimal value of 105 to 107° (38). Thus, the structure could represent a pre-Michaelis complex, and subsequent conformational change brings the aldehyde into the optimal orientation for nucleophilic attack. Alternatively, suboptimal carbonyl orientation could be an artifact induced by hydrogen bonding with Ser844, which cannot occur with Cys844 in the WT enzyme.

Although we have focused on the enzyme-ligand complexes, the glutamate complex structure also reveals the conformation of the ligand-free, oxidized PRODH active site, which potentially provides information about conformational changes associated with the binding of proline. Comparison of this structure with the PRODH Michaelis complex (THFA complex, PDB: 5KF6) implies that the binding of proline provokes a cascade of conformational changes involving rotations of Tyr485, Arg489, Glu492, and Leu449 (Fig. S5). The conformational change of Tyr485 is striking, with the hydroxyl group of the side chain moving 14 Å. Conformational differences in the FAD are also evident and imply a crankshaft rotation of the ribityl chain upon proline binding to the resting enzyme.

Similarly, the structures of the noncovalent GSAL complex and acyl-enzyme show the reduced, ligand-free PRODH active site. The FAD is highly bent in these structures, consistent with 2-electron reduction. Notable features include conformational disorder of two key substrate-binding residues, Arg489 and Tyr485. As mentioned above, both residues experience large conformational changes upon the binding of proline to the oxidized enzyme (Fig. S5). Interestingly, the ion pair gate is open in the noncovalent GSAL complex and closed in the acyl-enzyme. This result raises the possibility of long-distance communication whereby catalytic events in the GSALDH active site influence the conformation of the PRODH site.

The MD simulations provided insight into the dynamics of substrate channeling, which was not apparent from crystal structure snapshots. The simulations showed the intermediate P5C moving from the PRODH active site into a buried tunnel leading to the GSALDH active site. These results support the hypothesis from crystal structures that the tunnel functions as the substrate-channeling pathway. P5C never diffused into the GSALDH active site, suggesting a bottleneck at the entrance of the active site located ∼32 Å along the channeling path (shaded red zone in Fig. 8*A*). It is possible that opening of the bottleneck requires a longer timescale than was simulated. Alternatively, it may be that hydrolysis of P5C to GSAL—the true substrate of GSALDH—is required for entry into the GSALDH active site. If so, it is tempting to propose that hydrolysis occurs at the high occupancy P5C site at ∼17.5 Å along the channeling path.

The MD simulations also support the hypothesis that an ion pair serves as a gate between the PRODH active site and the tunnel. Both residues of the ion pair are identically present in all PutA and monofunctional PRODH structures, implying a conserved mechanism. Static crystal structures implied a simple two-state gating mechanism: The gate closes after the binding of proline and during the hydride transfer step and opens to release P5C (24, 26, 36, 37). The MD simulations suggest a more complicated mechanism. The gate was observed to repeatedly open and close, or open for extended times (μs), while P5C remained in the PRODH active site. Also, in some trajectories, the gate continued to open and close after P5C release. Nevertheless, the gate was always in an open state at the moment when P5C was released into the tunnel. These results suggest a revised gating mechanism in which gate status is not necessarily synchronized with P5C occupancy, and opening of the gate is necessary, but not sufficient for release of P5C.

## Experimental procedures

### Preparation of crystals

The *Escherichia coli* expression system and protocols for producing recombinant SmPutA (UniProt F7X6I3) were described previously (31, 39). The expressed protein contains an N-terminal 6X-histidine tag and tobacco etch virus protease site. The plasmid encoding site-directed mutant variant E810A was generated using the Agilent QuikChange II site-directed mutagenesis kit, and the mutation was verified by Sanger sequencing. The plasmid encoding C844S was generated by GenScript. The enzymes were expressed and purified as described previously for WT SmPutA. The histidine tag was removed from all enzymes during purification.

Crystals of the L-glutamate complex were obtained by soaking crystals of SmPutA grown using the procedure described previously (31, 39). The crystals were soaked in a cryo-buffer consisting of the reservoir supplemented with ∼16% (w/v) PEG-200 and 325 mM L-glutamate and then flash-cooled in liquid nitrogen.

The structures of the FADH^−^-proline covalent adduct, noncovalent GSAL complex, and acyl-enzyme covalent intermediate were determined from crystals of the C844S and E810A variants soaked in cryobuffers containing the substrates of the coupled PRODH-GSALDH reaction. The soaks were performed in the 4 × 6 sitting drop trays that were used for crystallization. The reaction soaking process was started by adding 6 μl of the reaction mixture to the drop containing the crystal to increase the working volume. In triplicate, an additional 2 μl of the reaction solution was added, the drop was mixed by pipette aspiration, and 2 μl of excess volume was removed from the drop and discarded. The soak times were varied as follows: ≤ 1 min, 5 min, 11 min, 1 h, 4 h, 12 h, 24 h, 36 h, 48 h, and 1 month. The sitting-drop crystal trays were sealed with clear tape between soaking time points. A total of 61 crystals were harvested for data collection. Specific conditions used for the reported structures are described next.

The structure of the FADH^−^-proline covalent adduct was obtained by soaking crystals of C844S with a cryo-buffer containing the substrates of the coupled PRODH-GSALDH reaction. The crystals were grown in sitting drops formed by combining 2 μl of ∼5 mg/ml C844S (incubated for ∼1 h with 10 mM NAD^+^) with 2 μl of a reservoir solution containing 14% (w/v) PEG-3350, 0.2 M ammonium sulfate, 0.1 M sodium formate, 0.1 M magnesium chloride, and 0.1 M Hepes at pH 8.0. The crystal soaking solution contained 40 mM L-proline, 1 mM Coenzyme Q_1_, 1 mM NAD^+^, 20% (w/v) PEG-3350, 0.1 M Hepes at pH 8.0, 0.25 M ammonium sulfate, 0.1 M sodium formate, 0.1 M magnesium chloride, and 20% (w/v) PEG-200. The reaction was allowed to progress for 24 h, after which the pale-yellow crystals (indicating substantial reduction of complexed flavin) were harvested and flash-cooled in liquid nitrogen.

The structure of the noncovalent GSAL complex was also obtained with the C844S variant. Crystallization and soaking were done as described in the preceding paragraph except that the reaction was run for only about ≤1 min before flash-cooling in liquid nitrogen.

The structure of the acyl-enzyme covalent intermediate was obtained with the E810A variant. Crystals of E810A were grown in sitting drops formed by combining 2 μl of ∼5 mg/ml E810A (incubated for ∼1 h with 10 mM NAD^+^) with 2 μl of a reservoir solution containing 20% (w/v) PEG-3350, 0.15 M ammonium sulfate, 0.1 M magnesium chloride, 0.1 M sodium formate, and 0.1 M Hepes at pH 8.0. Crystals of E810A were soaked in the reaction mixture described above for the FAD-proline/P5C covalent adduct for 24 h and then flash-cooled in liquid nitrogen.

### X-ray diffraction data collection and refinement

Shutterless X-ray diffraction data were collected at the Advanced Photon Source beamlines 24-ID-C/E and Advanced Light Source beamline 4.2.2. The data were processed with XDS (40) and Aimless (41) through the automated pipelines of the beamlines. The space group is *C*2 with a dimer in the asymmetric unit. This is the same crystal form reported previously for SmPutA (27, 28, 31, 39, 42). Data processing statistics are listed in Table 1.

Initial phases were calculated by Fourier synthesis using models derived from deposited structures of SmPutA (PDB: 5KF6 or 6X9A). Iterative cycles of refinement and model building were performed with Phenix (43) and Coot (44). Geometrical restraints for the noncovalent ligands GSAL and L-glutamate were obtained with Phenix eLBOW (45) using isomeric SMILES strings.

Geometrical restraints for the covalent adducts were generated with the QMR procedure in Phenix, v1.21.1-5286 (46); a schematic overview of this implementation is outlined in Fig. S6. To obtain optimized, QMR-derived restraints for the covalent adduct of the acyl-enzyme, a GSAL molecule was generated from Phenix eLBOW and placed into 2*F*_o_-*F*_c_ density using Coot. The coordinates of the protein structure model with the newly placed GSAL were saved and submitted to Phenix *ReadySet!* for optimal placement of hydrogen atoms. The model output of Phenix *ReadySet!* underwent a round of Phenix refinement after defining a link (*i.e.*, covalent adduct) between the CD atom of GSAL and the SG atom of Cys844. The model output of Phenix refinement and starting Phenix eLBOW ligand restraints was submitted as input to the command-line QMR module of Phenix. The GSAL molecule was selected as the ligand of interest, the default buffer radius around GSAL of 3.5 Å was used, and the Molecular Orbital PACkage (MOPAC) QM package was used for the calculation. The geometric restraints produced from the QMR calculation were used to perform subsequent real-space refinement of the covalent acyl-enzyme adduct into 2*F*_o_-*F*_c_ density within Coot, as well as multiple rounds of Phenix refinement. The riding hydrogen refinement model was used for all Phenix refinements.

QMR-derived restraints for the FADH^−^-proline covalent adduct were obtained and used in a similar fashion as described for the acyl-enzyme, except that the adduct was formed by patching L-proline onto the N5 of FADH^-^ (chain B) using the sketcher feature of the SwissSimilarity server (47, 48); this procedure provided the starting SMILES string for the initial round of Phenix eLBOW. QMR-derived restraints for FADH^-^ in chain A were also generated and plane angle restraints were adjusted in Phenix REEL (49) to match those of a 2-electron reduced FAD.

The structures were validated using MolProbity (50) and the wwPDB validation service (51). Modeling of ligands was validated with polder omit maps (52). Refinement statistics are listed in Table 1. Atomic coordinates and structure factor amplitudes have been deposited in the PDB under accession codes: 9C34 (FADH^−^-proline adduct), 9C35 (acyl-enzyme), 9C36 (GSAL complex), and 9BBO (L-glutamate complex).

### MD simulations

A monomer of SmPutA was simulated in explicit solvent. The monomer is thought to be the minimal active species of SmPutA based on small-angle X-ray scattering (39) and the fact that neither active site is located in the dimer interface. Briefly, the data showed that SmPutA exists in solution as a monomer-dimer equilibrium. At a concentration of ∼1 mg/ml (8 μM), SmPutA is predominantly monomeric, and the fraction of dimer increases to about 50% at 30 μM enzyme concentration. Considering that SmPutA enzyme activity assays are performed at enzyme concentrations less than 1 μM, the monomer is likely the form of the enzyme responsible for the observed catalytic activity (39). Therefore, all-atom MD simulations were performed on an SmPutA monomer.

The starting coordinates for the simulations were obtained from chain A of the C844S crystal structure, which has the PRODH product P5C positioned next to the *si* face of the reduced flavin (PDB: 9C34). Crystallographic waters and other solvent molecules were removed, and missing side chains were manually placed in Coot by inspection of the electron density (scrolled to a liberal 2*F*_o_-*F*_c_ value of 0.6σ), prioritizing favorable contacts and preferred rotamers. For extended regions of missing backbone atoms (*e.g.*, N and C termini, residues 79–82, 135–136), predicted atom positions from an AlphaFold 3 (53) model of SmPutA were patched into missing regions, and refinement into electron density was performed when possible (while preserving flanking residue crystallographic positions with the “Fix Atoms” tool in Coot).

Catalytic ligands from the crystal structure were retained in the system (P5C, reduced FAD, NAD^+^, and Mg^2+^) while L-proline in the secondary binding site was removed for simplification in simulating the product P5C release from the PRODH active site. Separate PDB files for P5C, FADH_2_, NAD^+^, and Mg^2+^ were uploaded to the molecular editor Avogadro v1.2.0 (54) for Mol2 file creation. Mol2 files for FADH_2_ and NAD^+^ were obtained with the correct protonation states for pH 7.2 (predicted by SwissParam (55, 56, 57), where the pyrophosphate for each dinucleotide was fully deprotonated), a Mol2 file for a zwitterionic P5C was created based on knowledge of the PRODH reaction mechanism, and a Mol2 file for Mg^2+^ was saved with its default representation in Avogadro. The prepared protein (above) was stripped of its refined hydrogens, combined with the new ligand Mol2 files, and submitted to the PDB2PQR server (58) for side chain protonation prediction at pH 7.2 (necessary for generating proper nomenclature of protonated glutamate, aspartate, and histidine residues). System parameterization and topology file generation was performed with the CHARMM General Force Field (CGenFF version 4.6–July 2022) (59, 60).

MD trajectories were prepared as follows. System solvation, equilibration, and simulation runs (61, 62, 63, 64, 65, 66, 67, 68, 69) were performed with the GROMACS 2022.5 package (70) with direct GPU communication enabled. The protein was centered in a rhombic dodecahedral box, in which a minimum 10 Å buffer was applied between the protein and the box edge. The TIP3P water model (71) was used for system solvation with 45,558 water molecules, and 9 Na^+^ ions were added for system neutralization. The total number of atoms was 155,488. Energy-minimization using the steepest descent algorithm of the solvated, neutralized system was performed until the maximum force was less than 10.0 kJ mol^−1^ nm^−1^. The system was subjected to a second round of energy-minimization, with inclusion of van der Waals forces using a switching function with inner and outer cutoffs of 1.0 nm and 1.2 nm, respectively, as well as a harmonic biasing force of 5000 kJ mol^−1^ between Mg^2+^ and an O-atom (O2A) of the pyrophosphate of NAD^+^ (which was maintained in all subsequent steps). NVT and NPT equilibration steps were performed as previously described (72). An initial, 2-μs production simulation was obtained after release of positional restraints on the NPT-equilibrated structure, using a 2-fs time step. Periodic boundary conditions were applied in all three dimensions. 20 parallel production runs, each spawned from the first energy-minimization (then each uniquely underwent the second round of energy-minimization and equilibration steps), were simulated for 2-μs. Thus, the total simulation time for the 21 combined production runs was 42-μs. Coordinates were saved every 1 ns for analysis. Data analysis of the MD trajectories was performed using MDAnalysis v2.8.0 (73, 74). The GetContacts (https://getcontacts.github.io/) software was used to assess residue contact frequencies. Plots were generated using the Matplotlib package v3.10.0 (75). PyMOL v3.1.3 (76) was used for molecular visualization, and the PyMOL plug-in CAVER v3.0.3 (77, 78) was used for tunnel analyses.

## Data availability

Atomic coordinates and structure factor amplitudes have been deposited in the PDB under accession codes: 9C34 (FADH^−^-proline adduct), 9C35 (acyl-enzyme), 9C36 (GSAL complex), and 9BBO (L-glutamate complex).

## Supporting information

This article contains supporting information (21, 79, 80).

## Conflict of interest

The authors declare that they have no conflicts of interest with the contents of this article.
