# Supplementary material for: Visualization of covalent intermediates and conformational states of proline utilization A by X-ray crystallography and molecular dynamics simulations[image]
Source: J Biol Chem. 2025 Jul 28;301(9):110532. doi: 10.1016/j.jbc.2025.110532 (PMC12398946; doi:10.1016/j.jbc.2025.110532)
Supplement: Supporting information [file mmc1.pdf]

SUPPORTING INFORMATION

Visualization of Covalent Intermediates and Conformational States of Proline Utilization A by X-ray Crystallography and Molecular Dynamics Simulations

David P. Buckley[1], Donald F. Becker[2], and John J. Tanner[1,3,*]

[1]Department of Biochemistry, University of Missouri, Columbia, Missouri 65211, United States

[2]Department of Biochemistry, Redox Biology Center, University of Nebraska, Lincoln, Nebraska 68588, United States

[3]Department of Chemistry, University of Missouri, Columbia, Missouri 65211, United States

[*]Corresponding author: Department of Biochemistry, University of Missouri, Columbia, MO 65211. Phone: (573) 884-1280. E-mail: tannerjj@missouri.edu.

Table of Contents

**Figure S1.** Crystallographic water molecules in the tunnel………………………….….	S2
**Figure S2**. Tunnel waters from molecular dynamics………………………………….…..	S3
**Figure S3.** Similarity of the $FADH^-$-proline covalent adducts from X-ray crystallography and electronic structure calculations. ………………………………………	S4
**Figure S4.** Comparison of the covalent adducts of $FADH^-$ with proline and thiazolidine-2-carboxylate …………………………………………………………….	S5
**Figure S5.** Conformational changes associated with the binding of proline…….………..	S6
**Figure S6.** Summary of Phenix QMR approach………………………………………....	S7
**References** …………………………………………………………………………	S8

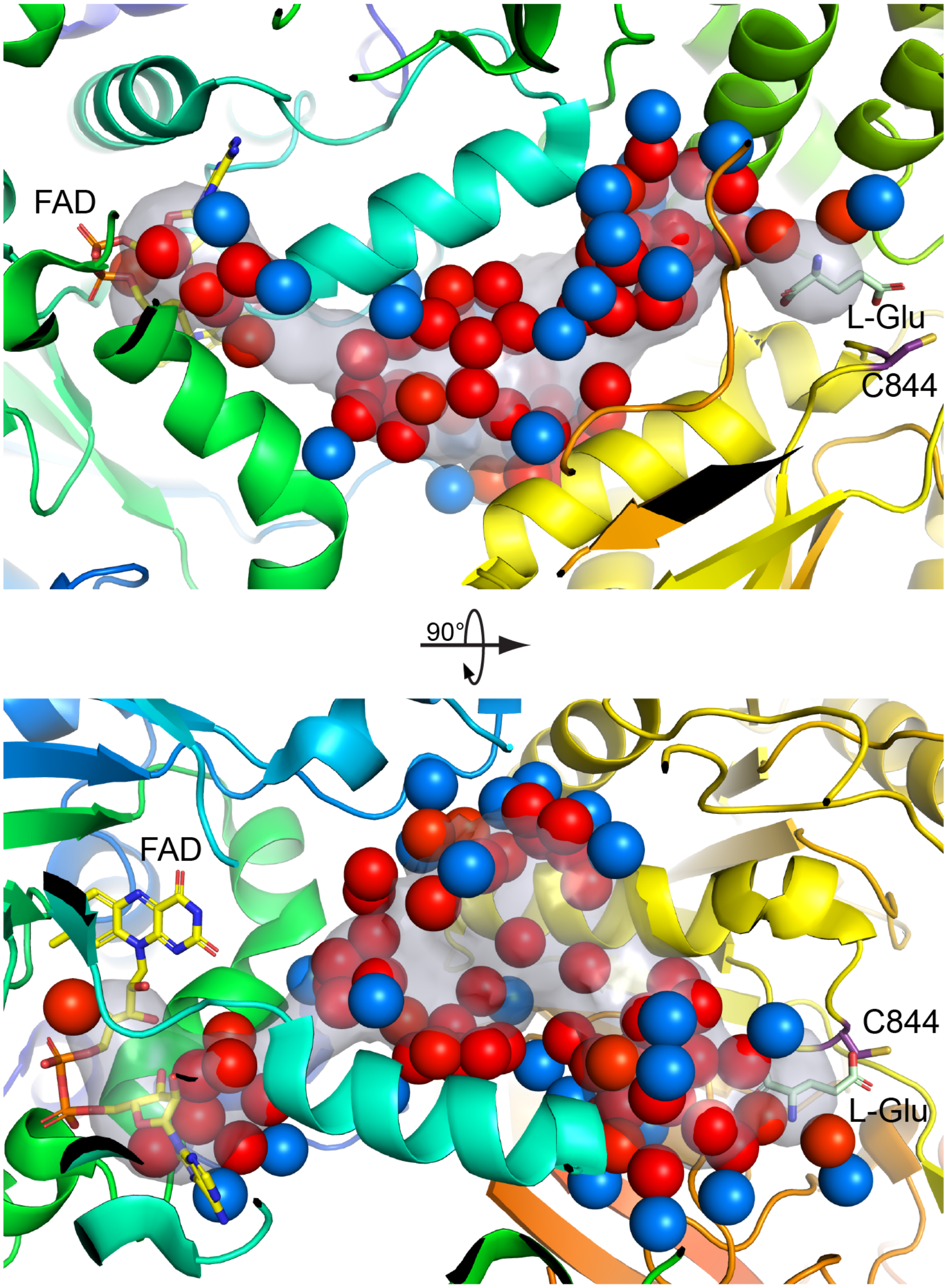

Figure S1. Crystallographic water molecules in the tunnel. Two views of water molecules in the tunnel of the glutamate complex (PDB: 9BBO). Red spheres represent crystallographic water molecules in the tunnel. Blue spheres represent crystallographic water molecules within 3.2 Å of the tunnel waters. The polypeptide chain is colored in a rainbow scheme with blue at the N-terminus and red at the C-terminus. The tunnel (semi-transparent gray) was calculated with the MOLEonline server (1).

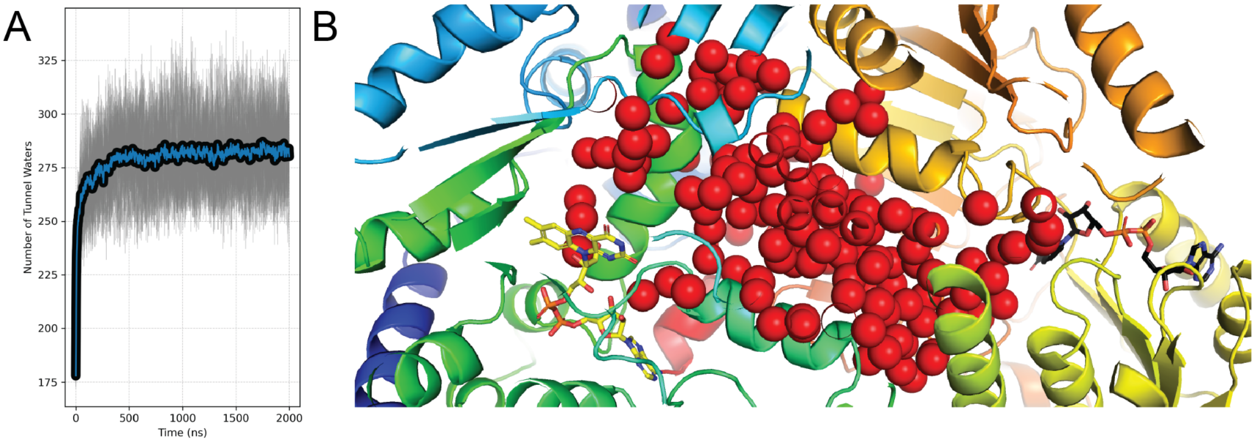

Figure S2. Tunnel waters from molecular dynamics. (A) Number of waters within 3.5 Å of tunnel-lining residues (i.e., residues identified by Caver Analyst v3.0 (2)) for each simulation (grey lines), with the running average number of waters shown in bolded black/blue lines. (B) Example MD snapshot of waters (red spheres) within the tunnel.

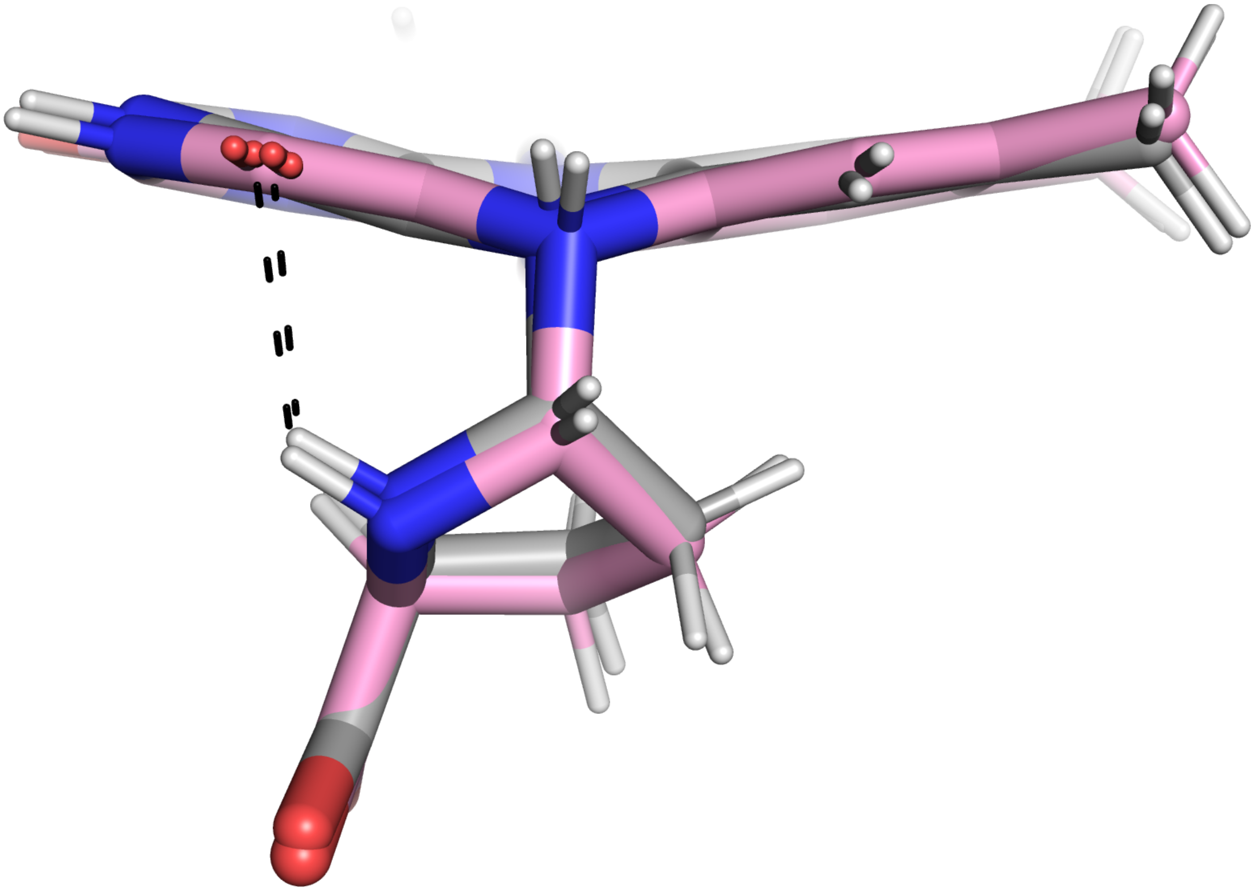

Figure S3. Similarity of the $FADH^{-}$-proline covalent adducts from X-ray crystallography and electronic structure calculations. The $FADH^{-}$-proline covalent adduct resolved from kinetic X-ray crystallography is shown in pink. The PC2 state predicted from electronic structure calculations is in gray (3). The dashes represent a potential hydrogen bond between the amino group of proline and O4 of FAD.

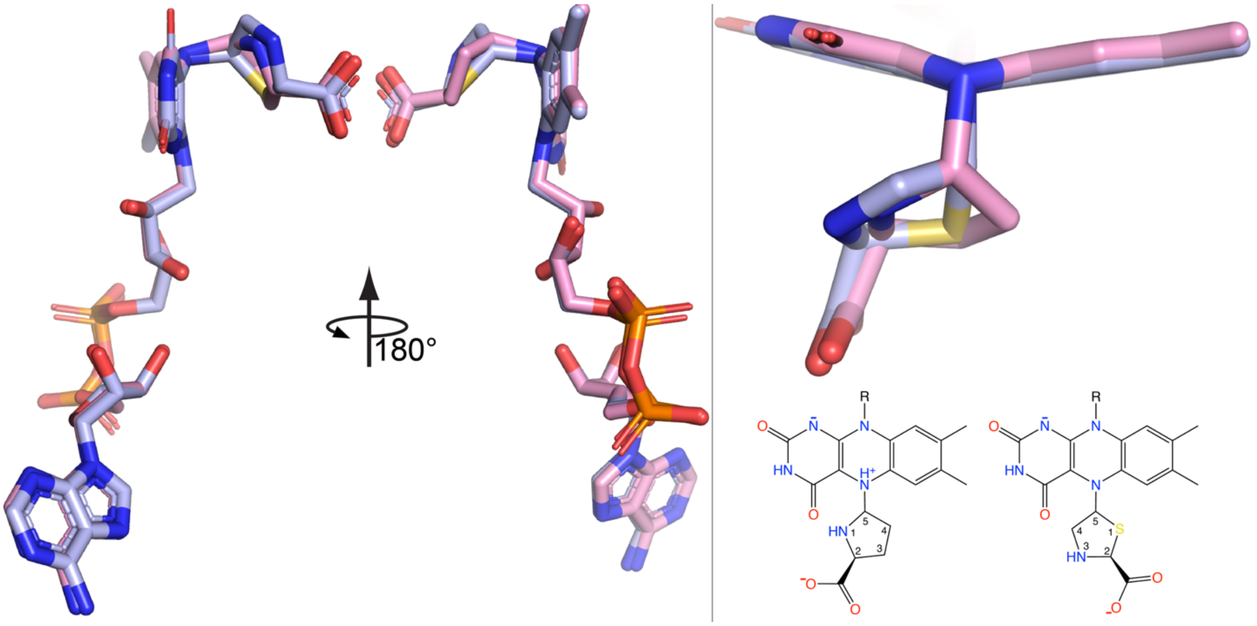

Figure S4. Comparison of the covalent adducts of $FADH^-$ with proline (pink, PDB: 9C34) and thiazolidine-2-carboxylate (blue, PDB: 6VZ9).

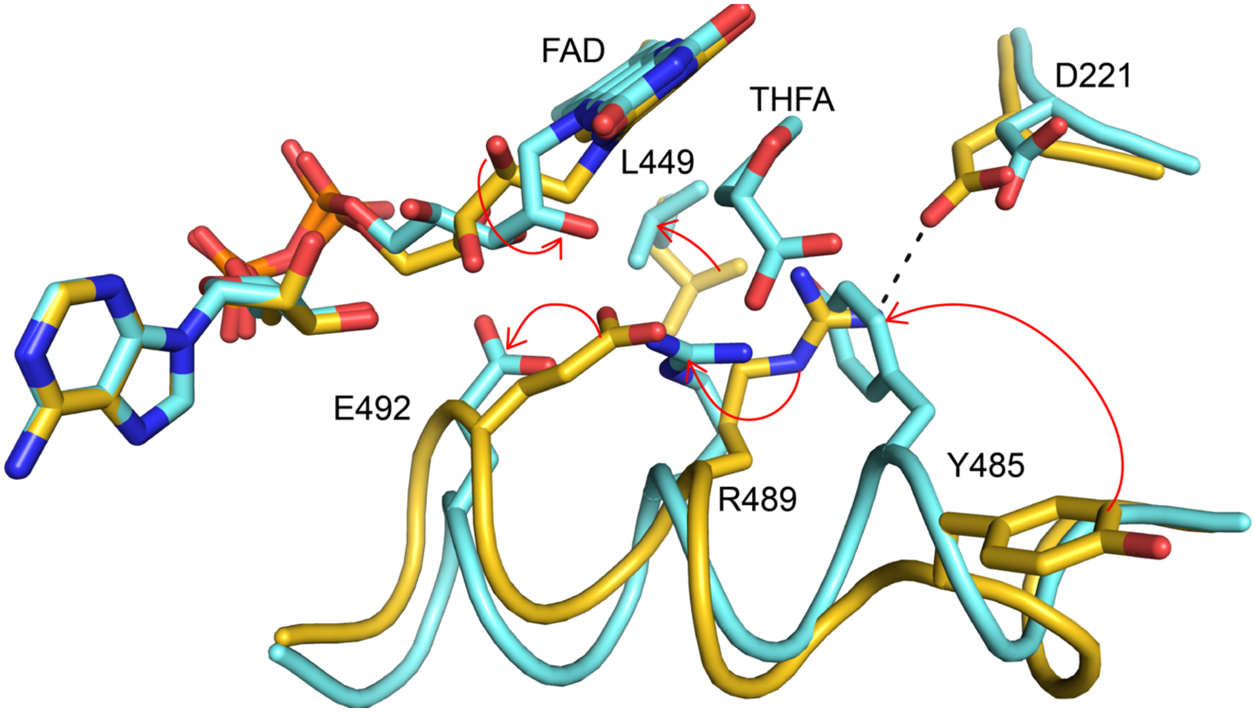

Figure S5. Conformational changes associated with the binding of proline to the PRODH active site. Comparison of the ligand-free PRODH active sites of the glutamate complex (gold, PDB: 9BBO) and the Michaelis complex with the proline analog THFA (aquamarine, PDB: 5KF6). The arrows indicate the direction of implied conformational changes.

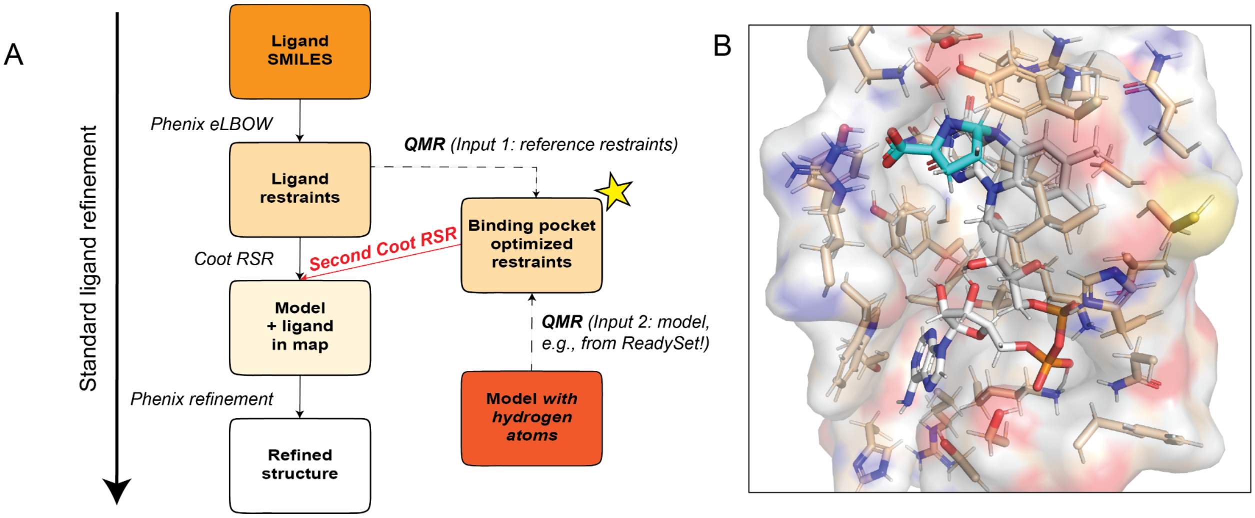

Figure S6. Summary of Phenix QMR approach. (A) Conventional ligand refinement pathway (solid lines) and inclusion of Phenix QMR-derived ligand restraint generation (dashed lines). RSR stands for real-space refinement. (B) Example output of Phenix QMR for $FADH^{-}$-proline adduct in the PRODH active site in which residues within 3.5 Å around the adduct were used in the QMR calculation.

References

1. Pravda, L., Sehnal, D., Tousek, D., Navratilova, V., Bazgier, V., Berka, K. *et al.* (2018) MOLEonline: a web-based tool for analyzing channels, tunnels and pores (2018 update) *Nucleic Acids Res* **46**, W368-W373
2. Jurcik, A., Bednar, D., Byska, J., Marques, S. M., Furmanova, K., Daniel, L. *et al.* (2018) CAVER Analyst 2.0: analysis and visualization of channels and tunnels in protein structures and molecular dynamics trajectories *Bioinformatics* **34**, 3586-3588
3. Yildiz, I. (2023) Computational insights on the hydride and proton transfer mechanisms of L-proline dehydrogenase *PLoS One* **18**, e0290901
